# 

**Published:** 1891-06

**Authors:** 


### MEETINGS--NOTICE.

### National Association of Boards of Dental Examiners.

The National Association of Boards of Dental Examiners will hold its annual meeting at Saratoga Springs, N. Y., on Monday August 3, 1891, at 10 a. m.

It is important that the Board of every State in the Union, that has a law regulating the practice of Dentistry, be as fully represented as possible. This Association has immense responsibility and it ought to have the benefit of the wisest and most discreet counsel possible. It is hoped that every Board having membership in this body will be represented and that those that have not yet become members will certainly do so at this meeting.

Matters of great interest and importance will come before the meeting. J. H. MARTINDALE, Sec'y.

Minneapolis, May 5, 1891.

### National Association of Dental Faculties.

The eighth annual meeting of the National Association of Dental Faculties will be held at Saratoga Springs, New York, on Saturday August 1st, 1891, at 10 o'clock a. m.

Applications for membership must be in the hands of the Executive Committee, Dr. J. Taft, Chairman, sixty days prior to the meeting.

Each delegate must be a member of the teaching faculty in the College he represents, and bring a certificate signed by the President (or Dean) and Secretary of his College, stating that he is authorized to act for them.

Delegates must be in attendance promptly at 10 A. M. on the day of meeting, in order that all the business may be concluded before the meeting of the American Dental Association, August 4th. J. D. PATTERSON, Sec'y,

Keith & Perry Building,

Kansas City, Mo.

### Missouri State Dental Association.

LOUISIANA, MO., July 7, 8, 9 and 10, 1891.

PARTIAL PROGRAMME.—PAPERS.

1. Dr. J. F. McWilliams, Mexico—"President's Address to State Association." Discussion opened by Dr. J. B. Newby, St. Louis.
2. Dr. J. D. Patterson, Kansas City—"Medication in the Treatment of Pulp Canals." Discussion opened by Dr. Theo. Stanley, Kansas City.
3. Dr. B. Q. Stevens, Hannibal—"The Preparation of the Mouth and Plate Work." Discussion opened by Dr. Wm. N. Morrison, St. Louis.
4. Dr. Frank Slater, Rich Hill—"The Devitalization and Removal of Tooth Pulps." Discussion opened by Dr. J. E. Crozier, Lee's Summit.
5. Dr. D. J. McMillen, Kansas City—"Crown and Bridge Work—Use and Abuse of Same." Discussion opened by Dr. T. M. Nickolson, Fayette.
6. Dr. A. H. Fuller, St. Louis—"Preparation of Cavities." Discussion opened by Dr. H. S. Lowry, Kansas City.
7. Dr. Wm. H. Eames, St. Louis—"Review of Dr. Miller's Work, with Personal Observation." Discussion opened by Dr.
8. Dr. Geo. L. Shepard, Sedalia—"Dental Fees." Discussion opened by Dr. L. A. Young, Neosho.
9. Dr. J. M. Austin, St. Joseph—"Report on New Appliances." Dr. A. J. Prosser, St. Louis—"Report on Clinics."
10. Dr. I. D. Pearce, Kansas City—"General Anæsthetics in Relation to Oral Surgery." Discussion opened by Dr. L. E. Day, Nevada.
11. Dr. E. E. Shattuck, Kansas City—"Regulating Appliances." Discussion opened by Dr. W. L. Reed, Mexico.

CLINICS.

1. Dr. J. D. Patterson, Kansas City—"Pulp Canal Filling."
2. Dr. J. Stephen Coyle, St. Louis—"Open-faced Crowns."
3. Dr. J. F. Hassell, Lexington—"Soft Gold Filling."

4. Dr. E. E. Shattuck, Kansas City, will exhibit "Regulating Appliances."

5. Dr. N. W. Pence, St. Louis—"Gold Filling, operator doing his own malleting."

6. Dr. K. R. Vaughan, Fulton—"Copper Amalgam, will also exhibit Bridge Work."

7. Dr. D. J. McMillen, Kansas City—"Soft Gold Filling."

8. Dr. B. Q. Stevens, Hannibal—"Amalgam Filling, to demonstrate packing and finishing. Will exhibit dies for striking up Face Teeth for Plate Work—Combination Fillings—Pulp Canal Impressions."

9. Dr. J. T. Fry, Moberly—"Compound Gold Filling."

10. Dr. J. B. Vernon, St. Louis—"Bicuspid Crown, Porcelain Face."

11. Dr. T. B. Carr, Stanberry—"Extracting."

12. Dr. E. B. Crane, California—"The Crane Vulcanizer, and a new method of moulding Rubber Plates. Will also show plates made the old way."

13. Dr. W. H. Buckley, Liberty—"Small Bridge Richmond Crown Attachments."

14. Dr. Geo. A. McMillen, Alton, Ills—"Will show the best Foot Blow Pipe for country dentists—those who have no Gas."

15. Dr. W. M. Carter, Sedalia—"Gold Filling, using Williams' Crystalloid Gold No. 3."

16. Dr. Wm. N. Morrison, St. Louis—"Lining Cavities with Gold and Platinum Folds, for Amalgam Fillings."

17. Dr. Wm. H. Eames, St. Louis—"New Form of Bridge Work, Teeth placed in position after Bridge is completed."

18. Dr. A. J. McDonald, Kansas City—"Open Faced Bicuspids for Bridge Work."

19. Dr. A. C. Griggs, Warrensburg—"Cleft Plate Appliances."

The usual railroad rates will be given, a fare and a third on the certificate plan. Be sure to get your receipt at starting point.

Hotel rates, $1.00 and $1.50 a day. A cordial invitation is extended to all reputable dentists; come and take part.

Dental depots will be represented. Electrical motors and appliances will be exhibited. Those having new appliances should send them to Dr. J. M. Austin, St. Joseph, Mo.

WM. CONRAD.
HENRY FISHER. } Executive Committee.
J. M. WHIPPLE.

### EDITORIAL.

### Michigan Meeting.

We are quite inclined to call special attention to the following notice of the Secretary of the Michigan State Dental Association. That body will hold its annual meeting, as will be seen, at Sault Ste Marie, Michigan. This place was selected at the last annual meeting because of the many attractions there at this time of the year. It affords a most agreeable and pleasant refuge from the great heat and oppressiveness of the more southern portions of the country, and in addition to this there are many things of very great interest to visit and see. We have no doubt there will be a large number in attendance. The trip will be a very pleasant one indeed, and will undoubtedly be made from Detroit by boat which will constitute a most delightful excursion.

For particulars inquire of the secretary or any officer of the society.

MICHIGAN DENTAL ASSOCIATION.—The thirty-sixth annual meeting of the Michigan Dental Association will be held at Sault Ste Marie, Mich., August 18th, 19th and 20th, 1891.

J. WARD HOUSE, Secretary.

GRAND RAPIDS, MICH.

### THE DENTAL REGISTER,

A MONTHLY MAGAZINE DEVOTED TO THE INTERESTS OF THE

### DENTAL PROFESSION.

*Terms Reduced to* $2.00 *Per Year. Single Copies,* 25 *Cents.*

### EDITED BY PROF. J. TAFT, D.D.S.

—AND—

### Published by SAM'L A. CROCKER & CO., Ohio Dental and Surgical Depot.

The volume commences in January and closes in December.

The REGISTER being issued monthly is always fresh, therefore Indispensable to the practitioner who desires to keep posted up with the new inventions and improvements which are daily developed. Neither expense nor effort will be spared to give value and variety to its contents. Articles will be illustrated with well executed engravings whenever they will add to the interest or assist to explanation.

Its extensive circulation and frequency of issue make it one of the BEST DENTAL ADVERTISING MEDIUMS in the United States.

All subscriptions must begin with January or July.

Local or special notices, five lines or less, will be inserted for $3 each insertion; over five lines, 50 cts. per line.

All advertising bills payable in advance, or at furthest, after the issue of the first number containing the advertisement. All transient advertisements to be paid for in advance.

☞CONTRIBUTIONS SOLICITED.

Exclusively Editorial Communications should be addressed to the Editor.

The latest number of the REGISTER may be ordered with or without other goods at any time, and all letters should be addressed to

